# Systems modelling ageing: from single senescent cells to simple multi-cellular models

**DOI:** 10.1042/EBC20160087

**Published:** 2017-07-11

**Authors:** Alvaro Martinez Guimera, Ciaran Welsh, Piero Dalle Pezze, Nicola Fullard, Glyn Nelson, Mathilde F. Roger, Stefan A. Przyborski, Daryl P. Shanley

**Affiliations:** 1 Institute for Cell and Molecular Biosciences, Campus for Ageing and Vitality, Newcastle Upon Tyne, U.K.; 2 The Babraham Institute, Babraham Campus, Cambridge, U.K.; 3 School of Biological and Biomedical Sciences, Durham University, Durham, U.K.; 4 The Bio-Imaging Unit, William Leech Building, William Leech Building, Newcastle Upon Tyne, U.K.

**Keywords:** aging, cell homeostasis, systems biology

## Abstract

Systems modelling has been successfully used to investigate several key molecular mechanisms of ageing. Modelling frameworks to allow integration of models and methods to enhance confidence in models are now well established. In this article, we discuss these issues and work through the process of building an integrated model for cellular senescence as a single cell and in a simple tissue context.

## Introduction

The process of ageing is multi-factorial and complex. Despite the complexity simple interventions such as inhibition of mTOR, activation of autophagy and supplementation with NAD are known to modulate the process of ageing with measurable benefits on mortality and lifespan [1]. To take full advantage of such findings, we need further detail on their mechanisms, to know how different interventions could be used in combination and at what doses. Systems modelling can support this effort. This becomes readily apparent when considering the properties of a biological system that need to be characterized for a full understanding. Firstly, the set of elements that compose the system. Secondly, the interaction map between the elements, i.e. which element interacts with which. Thirdly, the controlling nature of the interaction, for instance, whether the interaction is activatory or inhibitory. Fourthly, the dynamics of each interaction, whether the kinetics are rapid, slow, linear or non-linear. Consider a researcher with complete information on all of these properties of a given biological system stored for example as a spreadsheet of IDs and numerical values. Would the researcher then formulate a hypothesis by looking back and forth between a complex interaction map and columns of time course plots to try and guess how an intervention or perturbation into the system might manifest? Given the complexity of biological networks, it would not be feasible to mentally compute this sort of analysis or indeed extract as much information as possible from it. Computational models are an intuitive way of mapping information, whether data or knowledge, to a coherent framework in a way that is systematic and transparent. The result is a representation that allows incremental updating as new knowledge or data become available. Importantly, it allows the in-depth analysis to: (i) explore whether current knowledge is sufficient to explain observations and (ii) educate the intuition into how the biological system may behave and furthermore generate new predictions and hypotheses that can direct new experimental efforts.

Computational models have been developed to study individual mechanisms in the context of ageing [2]. Many other models have been developed outside this context but have obvious relevance to ageing, including NF-κB and inflammation [3]; p53 and DNA damage [4]; and mTOR and autophagy [5]. The homoeostasis of organisms depends on the integrity and interaction between such processes and integration of our knowledge presents an enormous challenge. The systems biology research community has developed standards for representing biochemical network models which are now widely adopted [6]. A key benefit is that existing models available in public repositories, such as Biomodels [7] and CellML [8], provide a resource that can be modified, extended and merged to address questions beyond their original focus. Thus, these integrative models can then serve to guide experimentation, explore the relative importance of different processes in different contexts, identify potential interventions and address fundamental issues such as maintenance of homoeostasis.

**Figure 1:**
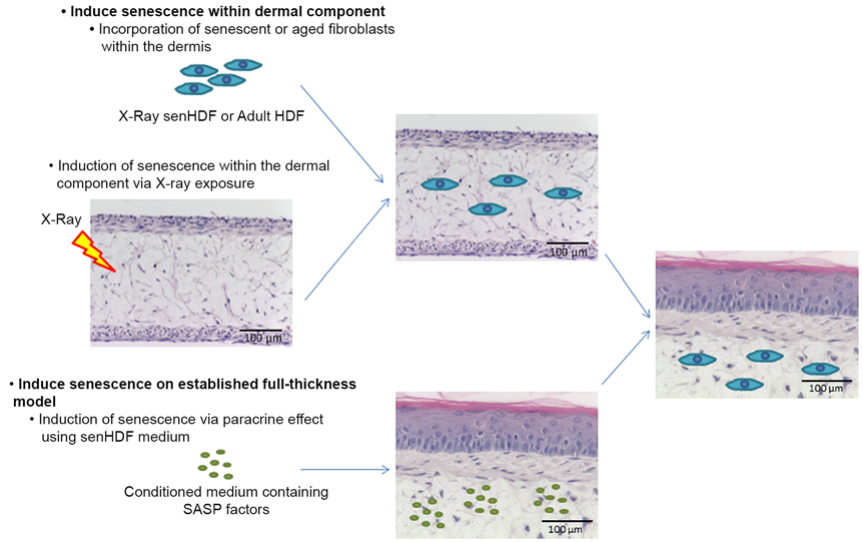
A 3D Alvetex-based skin model for studying cellular senescence. 3D Alvetex-based skin models can be adapted to study senescence either by inducing senescence within the dermal component or the established full thickness model.

In this article, we will focus on the process of modelling by working through a population dynamics study of senescent cells in a small lattice to represent a tissue. We will demonstrate the value of integrating different mechanisms; outline data and modelling issues that need to be addressed; and conclude with considering the specific challenge of cellular interactions that must be addressed to model ageing at the tissue level. The presence of senescent cells has been clearly shown to impact on whole organism ageing [9] and there is much interest in developing senolytics for their removal in late age [10]. It is likely that complex combinatorial interventions will be necessary and computational models provide a useful means to explore different treatment strategies. A computational model of at the tissue level requires knowledge and data on component cells, essential intracellular mechanisms that govern cellular state, and their interaction such as the bystander effect whereby senescent cells affect their neighbours [11]. We have useful *in vitro* cell-based models to study the dynamics of cell populations in a tissue-like environment. A notable example is for skin where senescence can be induced in 3D models grown on alvetex matrix: (i) directly in dermal fibroblasts or ii) in established dermal fibroblast and keratinocyte co-cultured models (Figure 1). Studying such *in vitro* models are essential for delivering the data required for the *in silico* models and validating the predicted outputs from such simulations.

### Development of computational models

**Figure 2:**
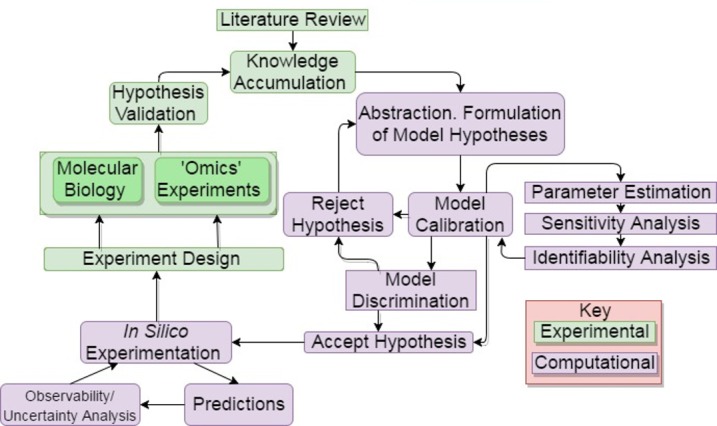
Integrated systems biology workflow. Systems modelling involves integration of methods from mathematics, computer science and molecular biology.

Computational models for ageing research are generally developed according to a series of stages shared with all systems modelling (Figure 2) [12]. Typically the background biology is thoroughly researched in the literature with due attention to pre-existing observations. Once an interesting biological question has been highlighted the major biological concepts are abstracted into a network representation of the system containing the key players and their interactions. A crucial aspect of the abstraction process is to minimize the biological detail needed to display the phenomenon of interest whilst maintaining enough detail for the model to retain predictive power.

In order for a model to have predictive power it must undergo a model calibration process whereby the model is trained on experimental observations. Two general schools of thought exist regarding model calibration, known as the Bayesian or frequentist approaches. Under the Bayesian regime statistical algorithms such as Markov Chain Monte Carlo (MCMC) can be used to maximize a likelihood function (maximum likelihood estimation, MLE) which measures the likelihood of observing experimental data given a model and parameter set. Frequentist methods, on the other hand, use numerical optimization techniques to estimate the true parameter set to a degree of confidence. Frequentist approaches are based on the central limit theorem in that parameter distributions tend to a normal distribution as the number of estimations increase. While neither approach is void of limitations, both methods contribute useful information to the parameter estimation process and can be used in conjunction with each other [13].

Model calibration in systems biology is particularly difficult due to a lack of detailed knowledge concerning the topological structure of a biological network. Further, a general lack of experimental data suitable for parameter estimation restricts the modelling process in multiple ways. For example, some model entities are impossible to measure and those that are tend to be measured with considerable experimental noise. In addition most experimental data in biology is measured in relative terms (e.g. Western blots or qPCR). Strategies that can be used to account for the use of relative data include using dimensionless model units (i.e. in arbitrary units like the experimental data) or by introducing nuisance variables such as scale factors and offset variables to relate relative to absolute terms. The problem with the former approach is that it does not describe the modelled process in physical terms while the problem with the latter approach is that it increases the dimensionality of an already large optimization problem. Optimization problems in systems biology are generally non-linear and occur in high dimensional parameter space with sloppy and non-identifiable parameters. The term sloppiness is used to describe the insensitivity of model predictions to large variations in certain combinations of model parameters even when informed by comprehensive datasets [14]. Non-identifiability refers to the inability of the defined optimization problem to locate unique parameter values [15]. It is customary to test for identifiability using one of many types of identifiability analyses such as the parameter profile likelihood approach [15]. Various software packages exist that compute the parameter profile likelihoods including Data2Dynamics [16], PottersWheel [17] and COPASI [18] with PyCoTools (https://github.com/CiaranWelsh/PyCoTools). Other routine types of analyses that are used in systems modelling include a global sensitivity analysis which measures a parameters impact on model output [19].

Once a model is calibrated, new experiments can be designed based on the model. Formal optimal experimental design methods can be used to maximize network coverage in an experiment and to resolve non-identifiabilities [20]. Model validation involves comparing simulation output to data that was not used to estimate parameters. A model is validated if simulation output and the validation dataset agree. If not then the model will need to be modified, and recalibrated with additional data if necessary. Mathematical procedures such as cross-validation [21] can be used to enhance statistical rigour and involves performing model calibration a number of times but rotating the validation dataset. Furthermore prediction and validation profile likelihood can be used to measure the uncertainly in model predictions and validations by propagating experimental errors through to model predictions [22]. In addition to the processes outlined here, other methods and approaches are available to deal with uncertainty quantification [23].

### Single cell model of irradiation-induced cellular senescence

Our starting point is a recently published model of cellular ageing developed to simulate irradiation-induced senescence [24]. This model was developed using an approach similar to that outlined in the preceding section. The underlying biology encompasses the interaction of the following systems: DNA damage signalling and repair; mitochondrial characteristics and turnover; insulin-mTOR network and oxidative stress response. Each of these systems have featured in separate computational studies [25–31] which formed part of the knowledge base on which the integrated model was developed. Sufficient pre-existing data were available to inform which components needed to be included in the model and the time frame for which further data needed to be gathered for formal parameter estimation. The following observables and the data generated provides some insight on the work involved: DNA damage, insulin-mTOR and stress response components were measured with a combination of Western blots and immunofluorescence; mitochondrial mass and membrane potential by live cell microscopy; superoxide and further measurement of mitochondria mass were measured with flow cytometry. Data were collected at selected time points to track the progression from a healthy to senescent cell. The model was used to explore the efficacy of interventions such as inhibitors of mTOR signalling, antioxidants and combinations of these interventions to slow or reverse the process of cellular senescence in a manner impossible to achieve with high enough throughput or at reasonable cost by *in vitro* methods. Potential intervention strategies identified by the model were then validated experimentally, but interestingly were found to be less effective once the senescent phenotype had fully developed. The likely cause was found to be that as senescence progressed the frequency of mitochondria fission events declined resulting in an extensive network of mitochondria inaccessible to mitophagy. This is an important finding that had so far been overlooked.

The accumulation of senescent cells in tissues with age is a complex process because the progression of cellular senescence involves interactions across multiple levels of biological organisation. At the molecular level, cellular senescence is triggered when DNA damage cannot be repaired, setting off a p21-mediated positive feedback loop through the further promotion of DNA damage by mitochondria [32]. Such a positive feedback loop drives and stabilizes the senescent state of the cell. At the cellular level, a senescent cell can further drive its own senescence progression or induce neighbouring cells to become senescent through the autocrine effects of the senescence-associated secretory phenotype (SASP) [33]. Both of these positive feedback loops have the potential to cause a runaway process that drives the establishment of senescent cell populations *in vivo*. However, negative regulation exists at molecular and cellular scales of biological organizations to counteract any runaway process promoted by the positive feedback loop architecture. At the molecular level, mitophagy removes dysfunctional mitochondria that produce the ROS needed to establish the senescent state [32,34]. At the cellular level, senescent cells can be recognized and destroyed by immune cells [33]. The accumulation of senescent cells with age can thus be regarded as a decrease in the effectiveness of negative regulation, an overflow of the operational mechanisms of negative regulation or both.

Progression of senescence within a tissue could thus be viewed as an interplay between a self-amplifying ability that drives accumulation and a counteracting negative regulation capacity. Any treatment that targets cellular senescence in tissues to improve health in an ageing context must consider the coupling of both of these feedback loops and their negative regulation across biological scales of space and time. As the molecular mechanisms behind senescence progression are further elucidated, new complexity will be added to this perspective. How can the increasing complexity of this nature be captured? Calibrated computational models such as that developed by Dalle Pezze et al. [24] have proven themselves to be an effective means of capturing the complex interactions that drive the progression of cellular senescence at the molecular level. Indeed, these models can serve as exploratory platforms for the *in silico* testing of novel combinatorial interventions and the generation of new hypotheses to direct and inform experimental efforts. These models can be coupled to frameworks that allow the progression of cell senescence at the molecular level to be mapped to individual cells interacting with each other in a tissue environment. This would be an example of a multi-scale model where molecular interaction networks are simulated for individual cells in an agent-based model.

### Multi-scale model of healthy and senescent cells

**Figure 3:**
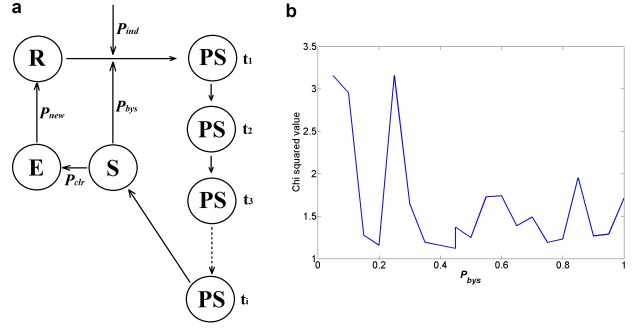
A simple multi-cellular model of cellular senescence. Developing a multi-scale computational model of cellular senescence progression in an arbitrary tissue. (**a**) State-transition interaction structure modelling the cellular scale. (**b**) Parameter fitting landscape for *P_bys_*.

It is a part of the complexity of many eukaryotic organisms that biological processes span levels of biological organization and different temporal scales. Computational modelling efforts have successfully tackled this level of complexity through the development of multi-scale models in a wide-variety of biological settings, including but not limited to, tumour growth [35], plant development [36], bone remodelling [37] and heart function [38]. Such efforts involve establishing couplings between computational frameworks modelling different scales through the use of formal methodologies [39]. To illustrate how such a multi-scale model of senescent cell accumulation in tissues might be formalized using available experimental observations, we build and simulate a simple multi-scale model of cellular senescence. We use Matlab (MathWorks Inc., Natick, MA, U.S.A., 2016) throughout. A cellular automaton framework [40] was adopted to model a population of interacting cells within a tissue (Figure 3a). The tissue is simulated as a regular lattice entirely occupied by resting cells (R). Such cells can transition to a series of other states throughout the simulation, namely, to a pre-senescence state (*PS*), to a senescent state (*S*) or to an empty state (*E*). At each time unit of simulation every cell is selected in turn and the following update rules are applied:

- If state is resting *(R)* then a transition to pre-senescence (*PS*) occurs with probability *P_ind_*
- If state is *(PS)* a transition to senescence *(S)* occurs after *t_i_* time units
- If state is *(S)* then neighbouring (*R*) cells change to *(PS)* with probability *P_bys_*
- If state is *(S)* and a minimum number of neighbours *S_n_* are in state *(S)* then a transition to empty *(E)* occurs with probability *P_clr_*
- If state is *(E)* then a transition to *(R)* occurs with probability *P_new_*

In this model, neighbouring cells are defined as those that are one coordinate unit away from the randomly selected cell in any dimension (Moore neighbourhood). The probability that senescence is induced *P_ind_* corresponds to a single stochastic run of the Dalle Pezze model of irradiation-induced senescence [24] for each resting cell at day 1. This aims to model an irradiation-induced senescence experimental protocol. However, a cell entering into a senescence program as a result of irradiation has a binary outcome, it will either become senescent or it will not. A given cell is defined to enter into a pre-senescent *PS* state if the stochastic simulation of the Dalle Pezze et al. model [24] yields a molecular abundance of four molecular markers of cellular senescence over a threshold defined by the average amount displayed by the entire irradiated cell population after 21 days. The four markers of cellular senescence were chosen to be p21, SA-β-Gal, ROS and DNA damage. Once the pre-senescence state (PS) is initiated, the transition time (*t_i_*) to a senescence state (S) is modelled to take 10 days. The value for the minimum number of neighbours *S_n_* was set to be half the number of neighbouring cells (i.e. 4 in the case of a 2D cell monolayer or 13 in the case of a 3D cell lattice) to reflect the idea that it is biologically more plausible for immune cells to be recruited to a group of senescent cells in tissue rather than a single individual cell within a tissue. The probability of clearance of senescent cells by the immune system *P_clr_* was assigned a value of 0.4 using *in vitro* data from a cytotoxicity study involving the 1:10 co-culture of senescent cells with natural killer cells [41].

Data derived for the *in vitro* progression of a senescence cell population was obtained from work published by Passos et al. [32] and used to perform a parameter estimation for the probability of the bystander effect *P_bys_*. This is possible since this data were derived from the *in vitro* culture of cells and so *P_new_* and *P_clr_* parameters can be set to zero in the parameter scan. Furthermore, the same irradiation protocol and cell line was used to collect the data that calibrated the Dalle Pezze model [24]. The multi-scale model was simulated for a 2D cell monolayer during the parameter scan procedure and the discrepancy between experimental measurements for a senescent cell population and simulation output was minimized using the chi-squared objective function. Parameter estimation reveals that multiple values of *P_bys_* fit the experimental data almost equally well (Figure 3b). This issue is due to a problem with identifiability and further data would be needed to resolve it. We assume that in a physiologically healthy setting *P_bys_* ≤ *P_clr_* since otherwise the occurrence of a single senescent cell at a young age would be able to drive a significant accumulation of senescent cells in a tissue. *P_bys_* was assigned a value of 0.2. The probability of a new cell occupying the empty space left by a cleared senescent state, *P_new_*, was set to 0.5 to maximize uncertainty at any given time unit. It is worth noting that cellular apoptosis was not included as a potential consequence of irreparable DNA damage because the 20G x-ray irradiation treatment on MRC5 cells, from which we derive our *P_bys_* and *P_ind_* values from, does not induce significant amounts of cellular apoptosis [24,42].

**Figure 4:**
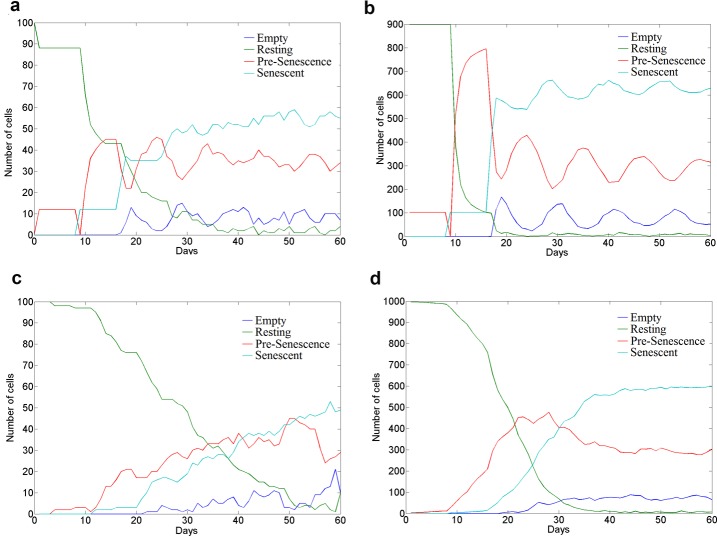
Simulation output from a multi-scale model of cellular senescence. Simulation output of the developed multi-scale model of cellular senescence progression in an arbitrary tissue. (**a**) Simulation output for irradiation-induced senescence in a 2D cellular grid. (**b**) Simulation output for irradiation-induced senescence in a 3D cellular grid. (**c**) Simulation output for stochastic-entry senescence in a 2D cellular grid. (**d**) Simulation output for stochastic-entry senescence in a 3D cellular grid. Parameters employed throughout all simulations correspond to *P_new_* = 0.5, *P_clr_* = 0.4, *P_bys_* = 0.2 and *P_ind_* corresponds to a single stochastic run of the Dalle Pezze et al. model [24]. 2D model simulations involve a regular grid of cells with dimensions 10 × 10. 3D model simulations involve a regular grid of cells with dimensions 10 × 10 × 10.

Model simulations are shown in Figure 4 for both irradiation-induced senescence (Figure 4a and b) and stochastic-entry into senescence (Figure 4c and d). The former case is simulated by a single stochastic run of the Dalle Pezze model [24] for every resting cell at day one. The latter case is simulated by simulating a single stochastic run of the Dalle Pezze et al. model [24] for any resting cell at any point in the simulation with probability 0.02. This aims to introduce temporal uncertainty as to when a given cell will experience a strong damage event that may cause it to enter senescence as a consequence. It is interesting to note that the model simulations converge to the same steady states of cellular populations whether senescence induction is through irradiation or stochastic entry or whether the simulation takes place in a 2D cell monolayer or a 3D cellular lattice. It can be appreciated how in the model of irradiation-induced senescence, an initial ∼10% of the cells become senescence as a result of the irradiation and this percentage is enough to eventually drive the whole tissue population into senescence (Figure 4c,d). This observation emphasizes the importance of the bystander effect as a molecular target to maintain tissue function with ageing. However, further simulations show that *P_bys_* determines the kinetics of senescence progression towards a steady state population that is actually determined by the *P_clr_* and *P_new_* parameters (data not shown). This would suggest targeted immunotherapy against senescent cells as a more effective strategy to reduce the accumulation of senescent cells with age than targeting the SASP.

It is worth noting that whilst the transition probabilities utilized are informed and constrained by experimentally derived observations, these are just working values and are likely to not only have different values in different biological contexts but also changing values with time. For instance, the decrease in immune function with age may mean a decreasing value for *P_clr_* with simulated time. Incorporating age-related molecular changes into the Dalle Pezze et al. model [24] could be used to examine how such alterations percolate across levels of biological organization and affect cell populations within a tissue through a potentially altered value of *P_ind_*. This case study aims to demonstrate how computational models can be formalized and informed from experimental observations to educate our intuition on how complex processes like ageing and senescence may unfold.

## Conclusion

We have provided an illustration of how tissue level models can be developed using an agent-based approach comprising of instances of well characterized cellular level models and clearly defined rules governing interaction between the cellular models. This work highlighted the need to acquire further data on cell–cell interactions and their mediators. We opted for the simple modelling framework of cellular automata so as not to distract from our main theme of incorporating knowledge and data within the models. Although transparent this framework suffers from problems such as scalability and computational efficiency. There are several options for multi-scale modelling that are available that may be explored for more substantial work in modelling ageing tissue. The use of finite state machines coupled – as here – with intracellular biochemical network models [43] is one possibility. A framework compatible with other tissue level modelling as, for example, in the Virtual Physiological Human project [44] would be particularly beneficial. Whatever the strategy it is important that the component cellular models are extensible to enable refinements such as that identified here where cellular senescence could also be enhanced to include other contributory mechanisms such as age-related changes in inflammation or reactive oxygen species scavenging.

## Summary

- The process of ageing is multifactorial and complex. Computational modelling provides a means to support research and identify potential interventions.
- There are a number of software tools and methods available for model development and fitting to data.
- Multiscale modelling frameworks enable the study of molecular and cellular mechanisms at the tissue and ultimately whole organism level.

## Abbreviation

mTOR: mammalian target of rapamycin
NAD: nicotinamide adenine dinucleotide
NF-kB: nuclear factor-κB
qPCR: quantitative polymerase chain reaction
ROS: reactive oxygen species
SASP: senescence-associated secretory phenotype

## References

[1] Lopez-Otin C., Galluzzi L., Freije J.M.P., Madeo F. and Kroemer G. (2016) Metabolic control of longevity. Cell 166, 802–821 27518560 10.1016/j.cell.2016.07.031

[2] Mc Auley M.T., Martinez-Guimera A., Hodgson D., McDonald N., Mooney K.M., Morgan A.E. (2017) Modelling the molecular mechanisms of aging. Biosci. Rep. 37, 1–20 10.1042/BSR20160177 PMC5322748 28096317

[3] Ashall L., Horton C.A., Nelson D.E., Paszek P., Harper C.V., Sillitoe K. (2009) Pulsatile stimulation determines timing and specificity of NF-κB-dependent transcription. Science 324, 242–246 19359585 10.1126/science.1164860 PMC2785900

[4] Purvis J.E. and Lahav G. (2013) Encoding and decoding cellular information through signaling dynamics. Cell 152, 945–956 23452846 10.1016/j.cell.2013.02.005 PMC3707615

[5] Dalle Pezze P., Ruf S., Sonntag A.G., Langelaar-Makkinje M., Hall P., Heberle A.M. (2016) A systems study reveals concurrent activation of AMPK and mTOR by amino acids. Nat. Commun. 7, 13254 27869123 10.1038/ncomms13254 PMC5121333

[6] Hucka M., Finney A., Sauro H.M., Bolouri H., Doyle J.C., Kitano H. (2003) The systems biology markup language (SBML): a medium for representation and exchange of biochemical network models. Bioinformatics. 19, 524–531 12611808 10.1093/bioinformatics/btg015

[7] Chelliah V., Juty N., Ajmera I., Ali R., Dumousseau M., Glont M. (2015) BioModels: ten-year anniversary. Nucleic Acids Res. 43, D542–D548 25414348 10.1093/nar/gku1181 PMC4383975

[8] Lloyd C.M., Lawson J.R., Hunter P.J., Nielsen P.F. (2008) The CellML model repository. Bioinformatics 24, 2122–2123 18658182 10.1093/bioinformatics/btn390

[9] Baker D.J., Childs B.G., Durik M., Wijers M.E., Sieben C.J., Zhong J. (2016) Naturally occurring p16Ink4a-positive cells shorten healthy lifespan. Nature 530, 184–189 26840489 10.1038/nature16932 PMC4845101

[10] Zhu Y., Tchkonia T., Fuhrmann-Stroissnigg H., Dai H.M., Ling Y.Y., Stout M.B. (2016) Identification of a novel senolytic agent, navitoclax, targeting the Bcl-2 family of anti-apoptotic factors. Aging Cell 15, 428–435 26711051 10.1111/acel.12445 PMC4854923

[11] Nelson G., Wordsworth J., Wang C., Jurk D., Lawless C., Martin-Ruiz C. (2012) A senescent cell bystander effect: senescence-induced senescence. Aging Cell 11, 345–349 22321662 10.1111/j.1474-9726.2012.00795.x PMC3488292

[12] Kitano H. (2002) Computational systems biology. Nature 420, 206–210 12432404 10.1038/nature01254

[13] Raue A., Kreutz C., Theis F.J. and Timmer J. (2013) Joining forces of Bayesian and frequentist methodology: a study for inference in the presence of non-identifiability. Phil. Trans. R. Soc. Lond. A 71, 20110544, doi: 10.1098/rsta.2011.0544 23277602

[14] Gutenkunst R.N., Waterfall J.J., Casey F.P., Brown K.S., Myers C.R. and Sethna J.P. (2007) Universally sloppy parameter sensitivities in systems biology models. PLoS Comput. Biol. 3, e189 10.1371/journal.pcbi.0030189 PMC2000971 17922568

[15] Raue A., Kreutz C., Maiwald T., Bachmann J., Schilling M., Klingmuller U. (2009) Structural and practical identifiability analysis of partially observed dynamical models by exploiting the profile likelihood. Bioinformatics 25, 1923–1929 19505944 10.1093/bioinformatics/btp358

[16] Raue A., Steiert B., Schelker M., Kreutz C., Maiwald T., Hass H. (2015) Data2Dynamics: a modeling environment tailored to parameter estimation in dynamical systems. Bioinformatics 31, 3558–3560 26142188 10.1093/bioinformatics/btv405

[17] Maiwald T. and Timmer J. (2008) Dynamical modeling and multi-experiment fitting with PottersWheel. Bioinformatics 24, 2037–2043 18614583 10.1093/bioinformatics/btn350 PMC2530888

[18] Hoops S., Sahle S., Gauges R., Lee C., Pahle J., Simus N. (2006) COPASI—a complex pathway simulator. Bioinformatics 22, 3067–3074 17032683 10.1093/bioinformatics/btl485

[19] Kent E., Neumann S., Kummer U., Mendes P. and (2013) What can we learn from global sensitivity analysis of biochemical systems? PLoS ONE 8, e79244 24244458 10.1371/journal.pone.0079244 PMC3828278

[20] Banga J.R. and Balsa-Canto E. (2008) Parameter estimation and optimal experimental design. Essays Biochem. 45, 195–210 18793133 10.1042/BSE0450195

[21] Hasdemir D., Hoefsloot H.C.J. and Smilde A.K. (2015) Validation and selection of ODE based systems biology models: how to arrive at more reliable decisions. BMC Syst. Biol. 9, 32 26152206 10.1186/s12918-015-0180-0 PMC4493957

[22] Kreutz C., Raue A. and Timmer J. (2012) Likelihood based observability analysis and confidence intervals for predictions of dynamic models. BMC Syst. Biol. 6, 120 22947028 10.1186/1752-0509-6-120 PMC3490710

[23] Geris L. and Gomez-Cabrero D. (eds) (2016) Uncertainty in Biology: a Computational Modelling Approach, Springer

[24] Dalle Pezze P., Nelson G., Otten E.G., Korolchuk V.I., Kirkwood T.B.L., von Zglinicki T. (2014) Dynamic modelling of pathways to cellular senescence reveals strategies for targeted interventions. PLoS Comput. Biol. 10, e1003728 25166345 10.1371/journal.pcbi.1003728 PMC4159174

[25] Dalle Pezze P., Sonntag A.G., Thien A., Prentzell M.T., Godel M., Fischer S. (2012) A dynamic network model of mTOR signaling reveals TSC-independent mTORC2 regulation. Sci. Signal. 5, ra25 22457331 10.1126/scisignal.2002469

[26] Dolan D., Nelson G., Zupanic A., Smith G.R. and Shanley D.P. (2013) Systems modelling of NHEJ reveals the importance of Redox regulation of Ku70/80 in the dynamics of DNA damage foci. PLoS ONE 8, e55190 23457464 10.1371/journal.pone.0055190 PMC3566652

[27] Dolan D.W., Zupanic A., Nelson G., Hall P., Miwa S., Kirkwood T.B.L (2015) Integrated stochastic model of DNA damage repair by non-homologous end joining and p53/p21-mediated early senescence signalling. PLoS Comput. Biol. 11, e1004246 26020242 10.1371/journal.pcbi.1004246 PMC4447392

[28] Lawless C., Jurk D., Gillespie C.S., Shanley D.P., Saretzki G., von Zglinicki T. (2012) A stochastic step model of replicative senescence explains ROS production rate in ageing cell populations. PLoS ONE 7, e32117 22359661 10.1371/journal.pone.0032117 PMC3281103

[29] Smith G.R. and Shanley D.P. (2010) Modelling the response of FOXO transcription factors to multiple post-translational modifications made by ageing-related signalling pathways. PLoS ONE 5, e11092 20567500 10.1371/journal.pone.0011092 PMC2886341

[30] Smith G.R. and Shanley D.P. (2013) Computational modelling of the regulation of Insulin signalling by oxidative stress. BMC Syst. Biol. 7, 41–41 23705851 10.1186/1752-0509-7-41 PMC3668293

[31] Sonntag A.G., Dalle Pezze P., Shanley D.P. and Thedieck K. (2012) A modelling–experimental approach reveals insulin receptor substrate (IRS)-dependent regulation of adenosine monosphosphate-dependent kinase (AMPK) by insulin. FEBS J. 279, 3314–3328 22452783 10.1111/j.1742-4658.2012.08582.x

[32] Passos J.F., Nelson G., Wang C., Richter T., Simillion C., Proctor C.J. (2010) Feedback between p21 and reactive oxygen production is necessary for cell senescence. Mol. Syst. Biol. 6, 347 20160708 10.1038/msb.2010.5 PMC2835567

[33] Perez-Mancera P.A., Young A.R. and Narita M. (2014) Inside and out: the activities of senescence in cancer. Nat. Rev. Cancer 14, 547–558 25030953 10.1038/nrc3773

[34] Correia-Melo C., Marques F.D., Anderson R., Hewitt G., Hewitt R., Cole J. (2016) Mitochondria are required for pro-ageing features of the senescent phenotype. EMBO J. 35, 724–742 26848154 10.15252/embj.201592862 PMC4818766

[35] Wang Z., Butner J.D., Kerketta R., Cristini V. and Dreisboeck T.S. (2015) Simulating cancer growth with multiscale agent-based modeling. Semin. Cancer Biol. 30, 70–78 24793698 10.1016/j.semcancer.2014.04.001 PMC4216775

[36] Muraro D., Larrieu A., Lucas M., Chopard J., Byrne H., Godin C. (2016) A multi-scale model of the interplay between cell signalling and hormone transport in specifying the root meristem of Arabidopsis thaliana. J. Theor. Biol. 404, 182–205 27157127 10.1016/j.jtbi.2016.04.036

[37] Colloca M., Blanchard R., Hellmich C., Ito K. and van Rietbergen B. (2014) A multiscale analytical approach for bone remodeling simulations: linking scales from collagen to trabeculae. Bone 64, 303–313 24713194 10.1016/j.bone.2014.03.050

[38] Quarteroni A., Lassila T., Rossi S., Ruiz-Baier R. (2017) Integrated Heart—coupling multiscale and multiphysics models for the simulation of the cardiac function. Comput. Meth. Appl. Mech. Eng. 314, 345–407

[39] Dada J.O. and Mendes P. (2011) Multi-scale modelling and simulation in systems biology. Integr. Biol. (Camb.) 3, 86–96 21212881 10.1039/c0ib00075b

[40] Machado D., Costa R.S., Rocha M., Ferreira E.C., Tidor B. and Rocha I. (2011) Modeling formalisms in Systems Biology. AMB Express 1, 45 22141422 10.1186/2191-0855-1-45 PMC3285092

[41] Krizhanovsky V., Yon M., Dickins R.A., Hearn S., Simon J., Miething C. (2008) Senescence of activated stellate cells limits liver fibrosis. Cell 134, 657–667 18724938 10.1016/j.cell.2008.06.049 PMC3073300

[42] Bluwstein A., Kumar N., Leger K., Traenkle J., Oostrum J., Rehrauer H. (2013) PKC signaling prevents irradiation-induced apoptosis of primary human fibroblasts. Cell Death Dis. 4, e498 23412390 10.1038/cddis.2013.15 PMC3734826

[43] Sütterlin T., Huber S., Dickhaus H. and Grabe N. (2009) Modeling multi-cellular behavior in epidermal tissue homeostasis via finite state machines in multi-agent systems. Bioinformatics 25, 2057–2063 19535533 10.1093/bioinformatics/btp361

[44] Cooling M.T., Nickerson D.P., Nielsen P.M.F., and Hunter P.J. (2016) Modular modelling with Physiome standards. J. Physiol. 594, 6817–6831 27353233 10.1113/JP272633 PMC5134412

